# Gender Differences in Genetic Risk Profiles for Cardiovascular Disease

**DOI:** 10.1371/journal.pone.0003615

**Published:** 2008-10-31

**Authors:** Kaisa Silander, Mervi Alanne, Kati Kristiansson, Olli Saarela, Samuli Ripatti, Kirsi Auro, Juha Karvanen, Sangita Kulathinal, Matti Niemelä, Pekka Ellonen, Erkki Vartiainen, Pekka Jousilahti, Janna Saarela, Kari Kuulasmaa, Alun Evans, Markus Perola, Veikko Salomaa, Leena Peltonen

**Affiliations:** 1 Department of Molecular Medicine, National Public Health Institute, Helsinki, Finland; 2 FIMM, Institute for Molecular Medicine Finland, Helsinki, Finland; 3 Department of Health Promotion and Chronic Disease Prevention, National Public Health Institute, Helsinki, Finland; 4 Department of Medical Epidemiology and Biostatistics, Karolinska Institutet, Stockholm, Sweden; 5 Indic Society for Education and Development, Nashik, India; 6 The Queen's University of Belfast, Belfast, United Kingdom; 7 Department of Medical Genetics, University of Helsinki, Helsinki, Finland; 8 Department of Human Genetics, Wellcome Trust Sanger Institute, Cambridge, United Kingdom; 9 The Broad Institute of MIT and Harvard, Boston, Massachusetts, United States of America; Erasmus University Medical Center, Netherlands

## Abstract

**Background:**

Cardiovascular disease (CVD) incidence, complications and burden differ markedly between women and men. Although there is variation in the distribution of lifestyle factors between the genders, they do not fully explain the differences in CVD incidence and suggest the existence of gender-specific genetic risk factors. We aimed to estimate whether the genetic risk profiles of coronary heart disease (CHD), ischemic stroke and the composite end-point of CVD differ between the genders.

**Methodology/Principal Findings:**

We studied in two Finnish population cohorts, using the case-cohort design the association between common variation in 46 candidate genes and CHD, ischemic stroke, CVD, and CVD-related quantitative risk factors. We analyzed men and women jointly and also conducted genotype-gender interaction analysis. Several allelic variants conferred disease risk for men and women jointly, including *rs1801020* in coagulation factor XII (HR = 1.31 (1.08–1.60) for CVD, uncorrected p = 0.006 multiplicative model). Variant *rs11673407* in the fucosyltransferase 3 gene was strongly associated with waist/hip ratio (uncorrected p = 0.00005) in joint analysis. In interaction analysis we found statistical evidence of variant-gender interaction conferring risk of CHD and CVD: *rs3742264* in the carboxypeptidase B2 gene, p(interaction) = 0.009 for CHD, and *rs2774279* in the upstream stimulatory factor 1 gene, p(interaction) = 0.007 for CHD and CVD, showed strong association in women but not in men, while *rs2069840* in interleukin 6 gene, p(interaction) = 0.004 for CVD, showed strong association in men but not in women (uncorrected p-values). Also, two variants in the selenoprotein S gene conferred risk for ischemic stroke in women, p(interaction) = 0.003 and 0.007. Importantly, we identified a larger number of gender-specific effects for women than for men.

**Conclusions/Significance:**

A false discovery rate analysis suggests that we may expect half of the reported findings for combined gender analysis to be true positives, while at least third of the reported genotype-gender interaction results are true positives. The asymmetry in positive findings between the genders could imply that genetic risk loci for CVD are more readily detectable in women, while for men they are more confounded by environmental/lifestyle risk factors. The possible differences in genetic risk profiles between the genders should be addressed in more detail in genetic studies of CVD, and more focus on female CVD risk is also warranted in genome-wide association studies.

## Introduction

According to world statistics for 2006, cardiovascular diseases (CVD) are responsible for 30% of all deaths globally, and are the leading cause of death amongst non-communicable diseases. Cardiovascular diseases are also responsible for 10% of the global burden of disease [1]. Differences in CVD incidence, complications and burden exist between men and women. Women are afflicted with cardiovascular disease at an older age than men, and many risk variables for coronary heart disease (CHD) and stroke have different distributions in men and women [2]–[7]. However, the differences in lifestyle factors do not fully explain the differences in CVD incidence between the genders [2]. Genetic factors also contribute to CHD and stroke susceptibility [8]–[10]. A recent large population-based prospective study suggested that heritability of ischemic stroke was greater in women than men [11]. Some of the traditional CVD risk factors also have high heritability [10], some of which show gender differences [12]. A large scale study of CVD traits in a Sardinian population showed that for several traits in which heritability estimates differed by gender, for example weight and hip circumference, the heritability was larger among women [13]. The evidence for gender differences in trait heritabilities implies possible gender-gene interaction in the etiology of these traits [12].

The effect of genetic variables on CHD and ischemic stroke has been studied for several decades, yet there are only a few consistent risk factors identified to date [10], [12], [14]–[19]. These genetic studies include few large scale candidate gene studies, as well as numerous smaller studies, and very recently several genome-wide association studies. Most of the large scale candidate gene studies published so far on CHD or stroke have performed combined analyses of both genders, using gender as a covariate [20]–[25]. In a Japanese case-control study of myocardial infarction, men and women were analyzed separately, and the significant results obtained for men and women were for different variants [26], indicating different genetic risk factors. In a large-scale genetic association study of the metabolic syndrome among CHD patients, McCarthy and colleagues identified several variants which displayed significant genotype-gender interaction [27]. In recent genome-wide association studies of CHD [14]–[17], [19] and ischemic stroke [28], the association results were reported for the combined study sample of both genders.

We estimated the effect of genetic variation on CHD, ischemic stroke and the composite end-point of CVD in two prospectively followed population cohorts. Our study had a case-cohort design on the FINRISK-92 and -97 cohorts participating in the MORGAM Project [29]. We selected 46 genes for study as putatively involved in cardiovascular pathobiology, based on their function, previous association with cardiovascular disease, and/or relevant phenotype in animal models. These genes represent a selected array of pathways, including lipid and energy metabolism, inflammation, coagulation, and thrombosis. We assessed the risk associated with common variation in each gene and CHD, ischemic stroke, and CVD while the cohort setting allowed us to control for classic CVD risk factors. We also assessed whether the variants affect relevant quantitative traits that are related to CVD risk: lipid and C-reactive protein (CRP) levels, blood pressure, body mass index (BMI), and waist/hip ratio (WHR). Our previous analysis of candidate genes like upstream stimulatory factor 1 (*USF1*) and Selenoprotein S (*SEPS1, SELS,* or *SELENOS*) mainly showed genetic effects in women [30], [31]. In this study, we therefore proceeded with a formal genotype-gender interaction analysis for all variants, and show that for several of the associated variants, there is evidence for statistical interaction between gender and genotype.

## Materials and Methods

### FINRISK cohort description

FINRISK surveys are carried out every 5 years to assess the prevalence and risk factors of CVD in Finland [32]. Baseline information on all randomly sampled individuals includes anthropometric measurements, serum lipids, blood pressure and questionnaire data on CVD risk factors. Information on fatal and non-fatal coronary and stroke events and all-cause mortality during the follow-up period is obtained from national registers. We utilized the FINRISK-92 cohort (n = 5999) and FINRISK-97 cohort (n = 8141), which have been followed up for 10 and 7 years, respectively. On these large cohorts, we conducted a case-cohort study, as previously described in detail [29], [31], [33]–[35]. The cohorts constituted respondents to surveys of independent random samples of the same geographically defined population. The resulting few overlaps were identified on the basis of personal ID codes, unique to every resident of Finland, and removed from the FINRISK-97 case-cohort set to ensure there was no overlap between the sets used for the analyses.

We initially studied the FINRISK-92 case-cohort set, which consisted of a total of 190 incident CHD cases, 66 incident ischemic stroke cases, 219 individuals with a history of either CHD or stroke event, 276 individuals who died during the follow-up, and a random sample (sub-cohort) of 398 individuals from the cohort. We also analyzed a second case-cohort set selected from the FINRISK-97 cohort, for genes associated with risk for CHD, ischemic stroke, the composite end-point of CVD or all-cause mortality, or strongly associated with quantitative traits in the FINRISK-92 case-cohort set. This sample included 210 incident CHD cases, 84 incident stroke cases, 436 individuals with a history of either CHD or stroke event, 352 individuals who died during the follow-up, and 407 sub-cohort individuals. The sub-cohort was a sex- and geographic-region stratified random sample, drawn from each of the original cohorts with unequal sampling probabilities so that the age distribution was similar to the cases. The selection procedure for the cases and the sub-cohort, and the exact diagnostic criteria used for CHD and ischemic stroke have been described in detail previously [29], [31], [33]–[35]. The case-cohort sets included in this study are described in Tables 1 and 2. All participants gave informed consent. In 1992 it was not yet customary to ask for a written consent, thus only oral informed consent exists for that survey. In 1997 a written informed consent was obtained from all survey participants. The law about the National Public Health Institute of Finland gives the Institute a possibility to also use the samples from the 1992 survey for public health research. The study was approved by the Ethics Committee of the National Public Health Institute of Finland and conformed to the principles expressed in the Declaration of Helsinki.

**Table 1:** Number of individuals in each of the case status categories in the FINRISK-92 and FINRISK-97 case-cohort study.

	FINRISK-92		FINRISK-97	
	women	men	women	men
TOTAL GENOTYPED	347	635	331	887
Sub-cohort^a^	114	284	87	320
Incident coronary cases^b^	52	138	51	159
Incident stroke cases^b^	32	34	21	63
Incident cardiovascular disease cases^b^	84	165	70	208
Death during follow up	91	185	91	261
Prevalent cardiovascular disease^c^	62	157	117	319
Incident CVD cases/baseline CVD cases in sub-cohort	7/3	37/29	10/7	30/59
Incident CVD cases/baseline CVD cases among deaths	11/7	48/47	25/14	60/79

a Random sample of the cohort, which included also some cases.

b These were incident during follow-up.

c Cardiovascular disease at baseline.

**Table 2:** Baseline characteristics according to case status, mean (std).

	Sub-cohort^a^				Incident cardiovascular disease cases				Prevalent cardiovascular disease cases			
	women		men		women		men		women		men	
	FR92	FR97	FR92	FR97	FR92	FR97	FR92	FR97	FR92	FR97	FR92	FR97
N	104	70	218	231	84	70	165	208	62	117	157	319
age at baseline examination (years)	55.9 (6.7)	62.9 (7.5)	52.8 (9.1)	60.0 (10.3)	54.6 (8.2)	63.1 (7.4)	54.2 (7.7)	62.0 (8.6)	57.1 (6.6)	60.8 (9.8)	56.7 (6.2)	62.2 (9.3)
age at first CVD event	NA	NA	NA	NA	60.3 (8.6)	66.7 (7.7)	59.5 (7.4)	65.6 (8.9)	NA	NA	NA	NA
Height (cm)	160 (7)	159 (6)	174 (7)	173 (7)	159 (6)	159 (6)	172 (6)	171 (7)	159 (6)	158 (7)	172 (7)	172 (7)
Weight (kg)	68.8 (12.9)	70.5 (11.6)	82.8 (12.9)	82.0 (13.1)	73.5 (12.8)	73.7 (12.3)	84.9 (13.7)	83.8 (12.2)	74.3 (15.2)	72.6 (14.0)	85.0 (13.7)	83.7 (12.7)
Body mass index (kg/m^2^)	27.1 (5.2)	27.7 (4.4)	27.4 (3.9)	27.5 (3.9)	29.1 (5.1)	29.3 (4.5)	28.7 (4.1)	28.6 (3.6)	29.4 (6.0)	28.9 (5.5)	28.7 (4.0)	28.5 (4.1)
Waist circumference (cm)	83 (12)	86 (11)	97 (11)	97 (11)	88 (12)	90 (12)	100 (11)	100 (10)	89 (14)	90 (13)	100 (10)	100 (10)
Hip circumference (cm)	104 (9)	104 (8)	102 (6)	102 (8)	105 (9)	106 (9)	103 (7)	103 (7)	107 (11)	105 (11)	104 (7)	103 (7)
Waist to hip ratio	0.80 (0.06)	0.83 (0.07)	0.95 (0.07)	0.94 (0.06)	0.84 (0.07)	0.84 (0.10)	0.97 (0.06)	0.97 (0.06)	0.82 (0.07)	0.85 (0.08)	0.97 (0.06)	0.97 (0.06)
Triglycerides (mmol/l)	1.36 (0.85)	1.58 (0.90)	1.80 (1.01)	1.77 (1.17)	2.04 (1.64)	1.91 (1.22)	2.37 (1.68)	1.98 (1.23)	1.74 (1.04)	1.76 (1.08)	2.15 (1.39)	2.06 (1.54)
Total cholesterol (mmol/l)	5.95 (0.98)	6.04 (1.10)	5.88 (1.02)	5.70 (1.10)	6.01 (1.28)	6.13 (1.17)	6.30 (1.10)	5.78 (1.02)	6.22 (1.07)	5.69 (1.12)	5.89 (1.10)	5.47 (0.99)
HDL cholesterol (mmol/l)	1.58 (0.38)	1.54 (0.38)	1.28 (0.35)	1.26 (0.33)	1.35 (0.38)	1.39 (0.37)	1.15 (0.31)	1.17 (0.28)	1.33 (0.37)	1.42 (0.37)	1.14 (0.29)	1.14 (0.32)
Non-HDL cholesterol (mmol/l)	4.37 (1.01)	4.50 (1.05)	4.60 (1.03)	4.44 (1.10)	4.66 (1.32)	4.74 (1.17)	5.14 (1.12)	4.60 (1.00)	4.89 (1.08)	4.27 (1.18)	4.74 (1.10)	4.33 (1.01)
Total to HDL cholesterol ratio	3.96 (1.14)	4.12 (1.15)	4.89 (1.40)	4.77 (1.42)	4.83 (1.83)	4.68 (1.37)	5.76 (1.58)	5.17 (1.44)	5.04 (1.81)	4.29 (1.55)	5.48 (1.76)	5.14 (1.77)
C-reactive protein (mg/l)	4.71 (10.08)	3.43 (7.76)	3.33 (8.43)	2.10 (3.45)	3.48 (4.81)	3.82 (5.74)	4.34 (5.48)	4.52 (11.71)	3.39 (5.45)	2.86 (4.06)	6.33 (10.11)	4.10 (6.19)
Diastolic blood pressure (mm Hg)^b^	84.7 (9.6)	82.8 (8.7)	86.3 (12.5)	86.5 (12.1)	85.8 (12.5)	84.2 (11.0)	88.1 (10.8)	87.1 (12.4)	85.5 (9.9)	83.3 (10.0)	85.6 (10.8)	84.5 (11.9)
Systolic blood pressure (mm Hg) b	145 (18)	148 (23)	141 (20)	146 (21)	148 (23)	151 (23)	149 (20)	150 (22)	145 (22)	145 (19)	144 (19)	146 (22)
% smokers	9.6	11.4	24.3	23.4	16.7	20.0	38.8	26.4	19.4	13.7	31.9	22.3
% high blood pressure	63.5	72.9	54.6	65.4	77.4	68.6	70.9	74.5	72.6	71.8	69.4	74.0
% diabetes	3.9	5.7	4.6	4.8	16.7	18.6	9.7	14.4	16.1	14.5	12.7	17.9
% lipid lowering drugs	1.0	2.9	2.8	3.9	3.6	2.9	3.6	7.2	9.7	18.8	15.9	22.3

a Sub-cohort individuals free of CVD at the end of follow up.

b Mean of two measurements.

### Quality control of DNA samples

We implemented several quality control measures to minimize errors associated with DNA sample handling and DNA quality, and excluded a total of 19 samples chosen as cases or in the sub-cohort. These 19 individuals are not included in Table 1. A gender-specific PCR test identified a total of 9 samples (0.4%) that had a different gender than expected, and they were subsequently excluded from the study. We also verified that the DNA sample was of good quality by testing five highly polymorphic microsatellite markers for each sample. In these analyses, one sample was found to be contaminated and was excluded. DNA samples with low DNA yield (<7.5 µg of genomic DNA) as measured by fluorescent label PicoGreen (Invitrogen, Carlsbad, CA, USA) were subjected to whole genome amplification before genotyping, followed by additional quality control checks [36]. A total of five samples were excluded due to biased whole genome amplification, and a further 4 samples were excluded due to extremely low quantities of DNA which was insufficient for whole genome amplification.

### Variant selection

For each gene, we aimed to genotype a set of variants that would capture the common variation present in the gene, as well as variants that have been previously associated with CVD or related traits. For the majority of the genes, haplotype-tagging single nucleotide polymorphism (SNP) variants were selected from the SeattleSNPs database (http://pga.gs.washington.edu/). The SeattleSNPs project has resequenced the genes using 24 Centre d'Etude du Polymorphisme Humain DNA samples, and tag SNPs have been selected using LDSelect, an algorithm that is based on the linkage disequilibrium (LD) statistic r^2^ [37]. We selected tag SNPs from each multi-SNP bin with a frequency >10%. For genes that were not included in the SeattleSNPs sequencing project, we selected variants from public databases (Celera, dbSNP), at approximately 5 kb distance from one another, giving priority to variants with known frequency information. Once HapMap phase I data were available, we selected additional variants to better capture the common variation in these genes. More detailed information about gene cladistics, sequence and haplotype structure information was available for apolipoprotein E *(APOE)*, lactase (*LCT)*, and lipin 1 (*LPIN1)*-genes, and here variant selection was based on previously published sequencing and haplotype analysis [38]–[41]. A full list of the variants selected for study and successfully genotyped (see below) is provided in Table S1.

### Variant genotyping

Variant genotyping was done using several genotyping platforms (Table S1). Approximately 5.5% of the genotypes were created with an in-house developed method of allele-specific primer extension on microarrays, as previously described [36]. Approximately 93.0% of the genotypes were produced with the MassARRAY System (Sequenom, San Diego, CA, USA), either with the homogeneous Mass Extension (hME) reaction or iPLEX reaction, using the protocols recommended by the manufacturer with these modifications: hME reactions were carried out with 5–7.5 ng of DNA and for the majority of the variants, the hME extension reaction was run using TERMIPol DNA polymerase (Solis Biodyne OÜ, Tartu, Estonia) [42] instead of ThermoSequenase (GE Healthcare Life Sciences, Chalfont St. Giles, UK). The two *APOE* variants that define the epsilon genotypes (*rs429358* and *rs7412*) were genotyped on the MassARRAY with a modified protocol as previously described [43] (full protocol available from authors upon request). Three of the variants were genotyped with other platforms: rs4340 was genotyped by a PCR assay followed by separation on 2% agarose gel with ethidium bromide staining and rs28665122 and rs3216183 were genotyped with TaqMan (Applied Biosystems, Foster City, CA) [30], [44]. For 100 samples where inadequate amount of genomic DNA was available, the DNA was amplified with GenomiPhi DNA amplification kit (GE Healthcare Life Sciences), as previously described [36].

Before genotyping the FINRISK case-cohort samples we genotyped all variants on 60 anonymous Finnish trio samples and 180 unrelated control samples. The FINRISK samples were genotyped in plates containing 2% negative control samples, 2% known duplicate samples, and 5% blind duplicate samples to allow assessment of genotyping quality. The disease status of each individual genotyped was unknown to the genotyping laboratory and samples from cases and sub-cohort individuals were distributed on the plates independently of the disease status. All genotypes were manually reviewed for various quality control aspects as previously described [36], [42], [45]. The genotyping success rate for each variant included in the analysis was >90%, with an average genotyping success of 95.3%. Among the 27,522 successful blind duplicate genotypic pairs, we detected 37 genotypic inconsistencies (99.87% concordance between genotypes). All variants included in analyses were in Hardy-Weinberg equilibrium (HWE) in the sub-cohort sample (p>0.01). A single Mendelian error was identified for 3 variants among the 60 trio samples (*rs1926446*, *rs3212478*, and *rs1081106*). However, since the genotypes for these variants were in HWE and no errors were detected among known and blind duplicates, these variants were included in the analysis.

### Statistical analysis

Genotype frequencies in sub-cohort individuals were tested for deviation from HWE using Pearson's chi-square test statistics with 1 degree of freedom for bi-allelic variants and 3 for three-allelic variants, applying a threshold of p<0.01. For variants in which one of the genotype groups had less than 5 individuals, HWE was calculated using an exact test. Allele segregation within trio families was analyzed with the PedCheck program [46]. Pair-wise LD between the variants in each gene, haplotype frequencies, and haplotype tags were assessed with Haploview software version 3.32 [47]. For variants in high LD with each other (r2>0.95), only one of the results is shown.

Time-to-event analysis was used to assess whether any of the tested allelic variants have effect on the incidence of CHD, ischemic stroke, or CVD. The effects under recessive, dominant and multiplicative models of individual variants were tested using the proportional hazards regression model where the case-cohort design was taken into account by applying a modification of the Prentice weighting [48], with the non-case sub-cohort members and sub-cohort cases before events weighted with the inverses of their individual inclusion probabilities to account for the over-sampling of cases[34]. Estimation of model parameters and standard errors was carried out in R statistical environment, using the coxph function of the package survival and its robust variance estimator. We adjusted for classic CVD risk factors: smoking, high density lipoprotein-cholesterol (HDL-C), non-HDL-cholesterol, history of diabetes, BMI, and hypertension, as well as geographic region (western Finland, northern Finland, and eastern Finland), and cohort (and gender for combined analysis in women and men). Age was used in the models as the time scale. We fitted two types of models. In the first model, men and women from both cohorts were analyzed jointly, as described above. In the second model, we carried out a test for genotype-gender interaction, defined as a departure from multiplicative, dominant or recessive model, using similar regression models and testing the null hypothesis of equality of genotype effect parameters between men and women. We report results in which the variant genotype specific p-value is ≤0.01 for either men or women. We verified that these results do not stem from a single cohort by testing the null hypothesis of equality of genotype effect parameters between FINRISK-92 and FINRISK-97 cohorts, using a similar regression model. For variants that conferred a risk at p<0.05 for CHD, we also studied the association in prevalent CHD cases (documented or self-reported myocardial infarction or unstable angina pectoris at baseline), using healthy sub-cohort subjects as controls. The analysis of prevalent cases was carried out using logistic regression, again with inverse sampling probability weighting, and using age, cohort and geographic region, and gender as covariates for the combined analysis of men and women. Analysis of haplotype effects was done for two variants of the F12 gene that were not in very high LD with each other and were both associated at p<0.01 with CHD and CVD. Haplotype analysis was done with an additive model, in which the common haplotype (containing the ‘non-risk’ alleles) was used as reference, and modeling an additive effect for the other haplotypes, in a weighted Cox proportional hazards model, applying the same weighting scheme and covariates that were used for single variant analysis, and using the PHREG procedure implemented in SAS version 9.1.3 SP4. Haplotype uncertainty was taken into account using multiple imputations, where a sample of haplotypes was obtained using Phase 2.1.1 software and the analysis was repeated for each sampled haplotype pair.

Additionally, we tested whether allelic variants were associated with quantitative traits measured at baseline in sub-cohort individuals without a history of CVD. The lipid variables studied were: serum total cholesterol, HDL-C, triglycerides, and low density lipoprotein-cholesterol (LDL-C). LDL-C was calculated from measured values of total cholesterol, HDL-C and triglycerides using Friedewald's formula and excluding individuals with triglyceride value >4.0 mmol/l. Additional variables studied were mean blood pressure (average of systolic and diastolic blood pressure, each value based on two subsequent measurements), high sensitivity CRP, BMI, and WHR. Association of the variants with baseline measurements was tested using standard linear regression, employing additive, dominant, and recessive models, while adjusting for cohort, age, geographic region, and gender. Tests for genotype-gender interaction, defined as a departure from additive, dominant or recessive model, were carried out using similar regression models and testing the null hypothesis of equality of genotype effect parameters between men and women. Individuals using lipid lowering medication were excluded from the analyses of lipid variables, and individuals using drugs for hypertension were excluded from the analysis of blood pressure. We used logarithmic transformation for CRP and triglycerides. We verified that the results reported do not stem from a single cohort by testing the null hypothesis of equality of genotype effect parameters between FINRISK-92 and FINRISK-97 cohorts, using a similar regression model.

For genes in which two or more variants (not in perfect LD) were associated at p<0.01 with a given quantitative trait, we also performed haplotype analysis to discern which allelic haplotype might be contributing to variation in the trait. Haplotype tagging variants were identified with the Haploview software version 3.32 using default settings. Analyses with the haplotype-tagging variants were performed with the haplo.stats package of the R statistical software [49], using the function haplo.glm with an additive model, and adjusting for age, cohort, geographic region and gender. The haplo.glm function estimates haplotype frequencies with the EM algorithm and calculates for each haplotype linear regression coefficient and p-value, comparing each haplotype to a base haplotype, defined as the most common haplotype. Rare haplotypes (frequency <0.05) were combined with the base haplotype for this analysis. The global p-value for haplotype effect coefficients was calculated for the null hypothesis of no effect for any haplotype.

For the initial analyses of the FINRISK-92 case-cohort alone, time-to-event analyses and quantitative trait analyses were done as previously described [30], [31], [33], analyzing women and men both separately and together. We did not perform formal gender-genotype interaction analysis or haplotype analysis at this stage.

In reporting the findings, we used a cut-off value of 0.01 for the p-values and reported uncorrected p-values. The cut-off value of 0.01 corresponds to posterior odds 6:1 of a finding being a true signal when we expect to see two signals among the 27 independent genes and our power is 70% (see The Wellcome Trust Case-control Consortium's 2007 paper for details) [19]. The effect of multiple testing was addressed with standard Q-Q-plots for the individual test statistics and with false discovery rate (FDR) analysis [50], [51]. The tail-area FDR statistic for a group of tests can be interpreted as the expected proportion of null results given the observed test statistics. The analysis was carried out using the R package “fdrtool” [52]. The method used for power simulations is described in more detail elsewhere [34]. The reported results are for both cohorts combined, for tests of the null hypothesis of no genotype effects (or no genotype-gender interaction) at 1% significance level. While simulating genotype-gender interaction we assumed no genotype effects for men while varying the effect for women.

## Results

### Study outline

The case-cohort sets from the FINRISK-92 (10 year follow up, 57,858 person-years) and FINRISK-97 (7 year follow up, 54,577 person-years) population cohorts [31] are presented in Tables 1 and 2. The list of genes and the number of variants successfully genotyped for each gene are presented in Table 3, and detailed information on all variants is presented in Table S1. In addition to known CVD candidate genes, we explored the effect of variation in the *LCT* gene on CVD risk and CVD related quantitative traits, because of previous findings of reduced triglyceride and cholesterol values in individuals with lactose malabsorption [53], [54]. We also studied one novel gene, apolipoprotein B mRNA editing enzyme (*APOBEC2*), which is located directly under a linkage peak (lod score of 4.44) for total cholesterol in our linkage study of 5775 individuals from twin families from the GenomEUtwin (www.genomeutwin.org). Individual results of the analysis for several of the genes have already been published: *USF1*, thrombomodulin (*THBD*), *SEPS1*, coagulation factor V (*F5*), protein C (*PROC*), and intercellular adhesion molecule 1 (*ICAM1*) [30], [31], [33], [44]. We include these genes here to provide a more complete picture of the observed difference in genetic susceptibility between men and women, and because formal genotype-gender interaction analysis was not reported for any of the genes in our previous publications.

**Table 3:** The genes included in the current study.

Inflammation & thrombosis	Gene symbol	Location	# variants	studied in both FR92 and FR97
carboxypeptidase B2 (plasma)	*CPB2*	13q14.12	10	yes
CD14 molecule	*CD14*	5q31.3	3	yes
coagulation factor II (thrombin)	*F2*	11p11.2	3	
coagulation factor II (thrombin) receptor	*F2R*	5q13.3	3	
coagulation factor V (proaccelerin, labile factor)	*F5*	1q24.2	21	yes
coagulation factor VII (serum prothrombin conversion accelerator)	*F7*	13q34	3	
coagulation factor X	*F10*	13q34	3	
coagulation factor XII	*F12*	5q35.3	3	yes
coagulation factor XIII, A1 polypeptide	*F13A1*	6p25.1	9	yes
C-reactive protein, pentraxin-related	*CRP*	1q23.2	6	yes
fibrinogen alpha chain	*FGA*	4q32.1	5	yes
fibrinogen beta chain	*FGB*	4q32.1	5	yes
fibrinogen gamma chain	*FGG*	4q32.1	4	yes
integrin, alpha 2 (CD49B, alpha 2 subunit of VLA-2 receptor)	*ITGA2*	5q11.2	6	
integrin, beta 3 (platelet glycoprotein IIIa, antigen CD61)	*ITGB3*	17q21.32	3	
intercellular adhesion molecule 1 (CD54), human rhinovirus receptor	*ICAM1*	19p13.2	6	yes
interleukin 1, alpha	*IL1A*	2q13	2	
interleukin 1, beta	*IL1B*	2q13	4	
interleukin 10	*IL10*	1q32.1	4	
interleukin 6 (interferon, beta 2)	*IL6*	7p15.3	6	
lectin, mannose-binding, 1	*LMAN1*	18q21.32	6	
lymphotoxin alpha (TNF superfamily, member 1)	*LTA*	6p21.33	4	yes
plasminogen activator, tissue	*PLAT*	8p11.21	4	
protein C (inactivator of coagulation factors Va and VIIIa)	*PROC*	2q14.3	6	yes
selectin E (endothelial adhesion molecule 1)	*SELE*	1q24.2	3	
selectin L (lymphocyte adhesion molecule 1)	*SELL*	1q24.2	6	
selectin P (granule membrane protein 140kDa, antigen CD62)	*SELP*	1q24.2	1	
selenoprotein S	*SEPS1*	15q26.3	6	yes
serpin peptidase inhibitor, clade E (nexin, plasminogen activator inhibitor type 1), member 1	*SERPINE1*	7q22.1	6	yes
thrombomodulin	*THBD*	20p11.21	9	yes
tumor necrosis factor (TNF superfamily, member 2)	*TNF*	6p21.33	2	yes
vascular cell adhesion molecule 1	*VCAM1*	1p21.2	4	
**Lipids & energy**				
apoliprotein A-V	*APOA5*	11q23.3	6	yes
apoliprotein E	*APOE*	19q13.32	7	yes
forkhead box C2 (MFH-1, mesenchyme forkhead 1)	*FOXC2*	16q24.1	4	
lactase	*LCT*	2q21.3	5	yes
lipin 1	*LPIN1*	2p25.1	7	yes
neuropeptide Y	*NPY*	7p15.3	4	yes
thioredoxin interacting protein	*TXNIP*	1q21.1	3	
upstream stimulatory factor 1	*USF1*	1q23.3	6	yes
**Others**				
5,10-methylenetetrahydrofolate reductase (NADPH)	*MTHFR*	1p36.22	3	
angiotensin I converting enzyme (peptidyl-dipeptidase A) 1	*ACE*	17q23.3	4	yes
angiotensin II receptor, type 1	*AGTR1*	3q24	7	yes
apolipoprotein B mRNA editing enzyme, catalytic polypeptide-like 2	*APOBEC2*	6p21.1	6	yes
fucosyltransferase 3 (galactoside 3(4)-L-fucosyltransferase, Lewis blood group)	*FUT3*	19p13.3	3	yes
klotho	*KL*	13q13.1	9	yes

**Figure 1:**
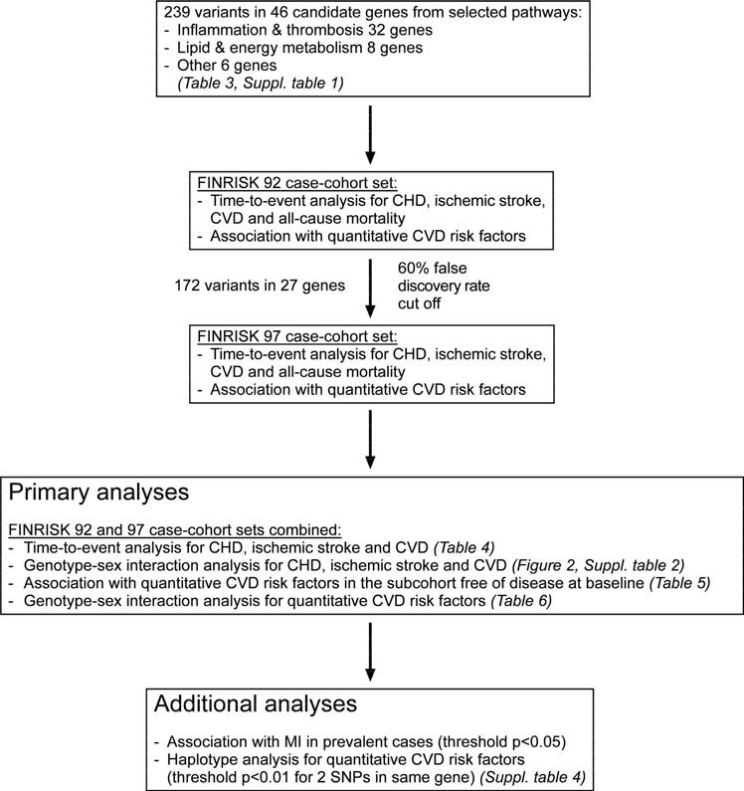
Study outline.

The study outline is presented in Figure 1. Initially, we studied the 46 genes in the FINRISK-92 case-cohort set. We selected for further study in the FINRISK-97 sample 27 genes in which one or more variants showed an association with CHD, ischemic stroke, CVD, total mortality, or any of the quantitative traits in the FINRISK-92 cohort, either in women or men separately, or in combined analyses. The selection criterion was 60% FDR. A total of 172 variants were thus typed also in the FINRISK-97 case-cohort samples, as indicated in Table 3 and Table S1, and analyzed using the combined FINRISK-92 and FINRISK-97 case-cohort sets. Power simulations are presented in Figures S1 and S2. For time-to-event analysis (Figure S1), the combination of the two cohorts has a 88% power to detect a dominant gene main effect on CVD risk of 1.8 in men at p = 0.01, a 39% power to detect a similar effect in women, and 96% power to detect this effect size when analyzing women and men together, given a risk allele frequency of 0.2 assuming a proportional hazards model. For a higher allele frequency the power is somewhat higher. For gene-gender interaction analysis, our study sample has power to detect only large differences in risk effects at p = 0.01, for example 38% power to detect a difference of HR = 1.0 versus HR = 1.8 for allele frequency of 0.4. For quantitative traits (Figure S2), combining both cohorts provides a power of 75% for detecting a 0.3 standard deviation difference at allele frequency of 0.2 in men at p = 0.01, while the power is much lower for the smaller study sample of women. For gene-gender interaction analyses the power is >85% only for large differences in the effects, for example no effect in men and a coefficient of 0.6 in women.

### Time-to-event analysis results

#### Analysis of both genders jointly

Time-to-event analysis was used to assess the association between variants and CHD, ischemic stroke and the composite end point of CVD. Results with p≤0.01 from combined analysis of both cohorts and both genders are shown in Table 4. The estimated FDR for the set of all association tests (including tests for quantitative traits) with p≤0.01 is 53%. These analyses identified variants in angiotensin II receptor type 1 (*AGTR1*), *APOE*, carboxypeptidase B2 (*CPB2*), and coagulation factor XII (*F12*) as conferring risk of CHD. The two variants of the *F12* gene also conferred risk of CVD, as did one variant of fibrinogen alpha chain (*FGA*) gene. Haplotype analysis for the two F12 variants, *rs4976691* and *rs1801020*, in which carriers of the specific ‘risk’ haplotypes (CA, CG, or GA for *rs4976691* and *rs1801020*, respectively) were compared to individuals homozygous for the non-risk haplotype GG did not reveal stronger association with CHD or CVD than analysis of single variants. For ischemic stroke, only one *SEPS1* variant, *rs7178239*, was associated at p≤0.01 in the combined analysis of both genders, but only the women contributed to this effect (see below). The most consistent result was for CHD association with the *F12* variant *rs1801020* (men and women combined, p = 0.005 for additive model), which also conferred risk at the p<0.05 level for CHD in both women and men when analyzed separately. The rest of the variants showed association at p<0.05 level in only one gender. We tested whether the results were driven by only one of the cohorts by assessing genotype-cohort interaction, and observed no interaction at p<0.05, suggesting that the results are similar in both cohorts. Variant *rs440446* of *APOE* showed association at p<0.05 also in both cohorts separately, while the rest of the variants showed association at p<0.05 in one cohort only, though a similar trend was observed in the other cohort.

**Table 4:** Results with p≤0.01for coronary heart disease and cardiovascular disease for the variants studied, analyzing men and women together.

phenotype	Gene	Variant rs#	Risk allele	Minor allele	Additive model^a^		Dominant model^a^		Recessive model^a^		Minor allele frequency	
					P	Hazard ratio (95% CI)	P	Hazard ratio (95% CI)	P	Hazard ratio (95% CI)	cases	controls
Coronary heart disease	*AGTR1*	*rs388915*	G	G	0.2	1.18 (0.92–1.51)	0.7	1.06 (0.80–1.40)	**0.008**	2.23 (1.23–4.06)	0.228	0.198
	*APOE*	*rs440446*	C	C	**0.003**	1.39 (1.12–1.72)	**0.004**	1.50 (1.13–1.99)	0.09	1.52 (0.93–2.48)	0.323	0.282
	*CPB2*	*rs7336399*	A	G	**0.005**	1.35 (1.09–1.67)	0.2	1.33 (0.90–1.97)	**0.003**	1.55 (1.16–2.08)	0.349	0.415
	*F12*	*rs4976691* b	C	C	**0.009**	1.32 (1.07–1.63)	0.04	1.33 (1.01–1.77)	0.02	1.69 (1.10–2.59)	0.328	0.286
	*F12*	*rs1801020*	A	A	**0.005**	1.36 (1.10–1.69)	**0.004**	1.51 (1.14–2.00)	0.3	1.34 (0.80–2.24)	0.275	0.228
Cardiovascular disease	*F12*	*rs4976691* b	C	C	**0.006**	1.31 (1.08–1.59)	0.02	1.36 (1.05–1.76)	0.04	1.54 (1.02–2.31)	0.327	0.283
	*F12*	*rs1801020*	A	A	**0.006**	1.31 (1.08–1.60)	**0.003**	1.48 (1.14–1.92)	0.6	1.15 (0.70–1.88)	0.271	0.228
	*FGA*	*rs2070018*	A	G	**0.01**	1.45 (1.09–1.91)	0.3	1.68 (0.59–4.82)	**0.009**	1.49 (1.10–2.02)	0.128	0.156

a Time-to-event analysis, showing results for risk allele for the best model. Covariates used in analysis: geographic region, cohort, HDL-cholesterol, non-HDL cholesterol, body mass index, hypertension, smoking status, history of diabetes, and gender. P-values≤0.01 are in bold-face. P-values are not corrected for multiple testing.

b Results for *F12 rs4976691* are similar to those for *rs1787603*2, since they are in almost perfect LD (r^2^>0.96).

#### Gender-genotype interactions

**Figure 2:**
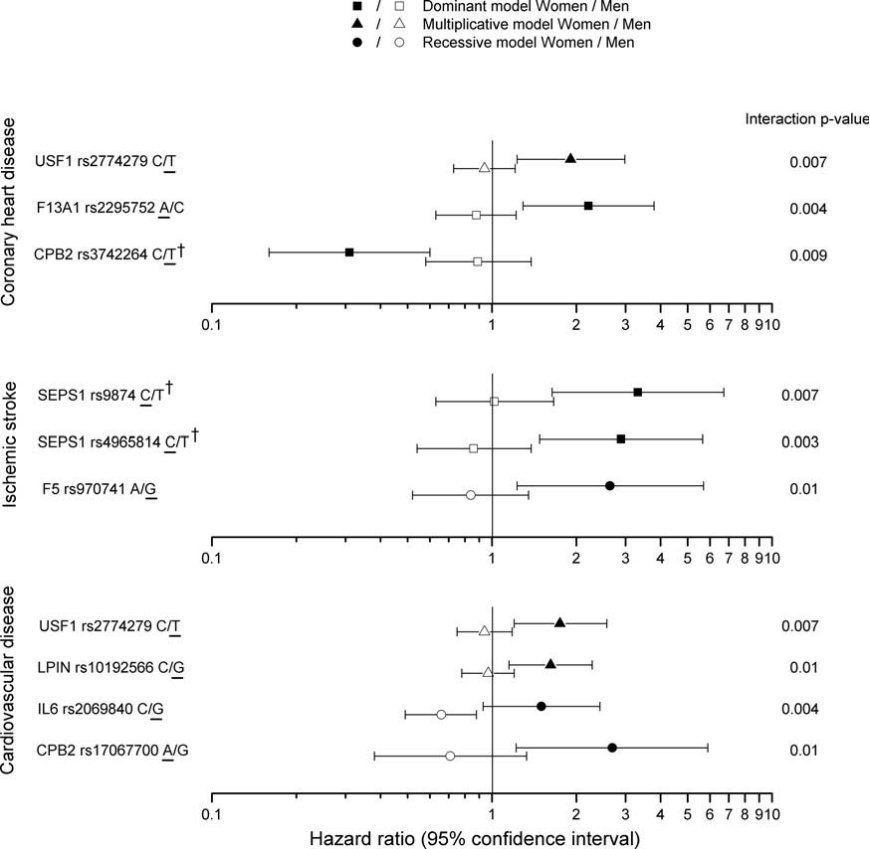
Gender-specific association between variants and coronary heart disease, ischemic stroke, and cardiovascular disease. Results for gender-genotype interaction at p<0.05, and association in either women or men were at p≤0.01 (uncorrected p-values). Allele information: allele 1/allele 2, the minor allele is underlined. Multiplicative model: 11>12>22, dominant model: 11+12 vs 22, recessive model: 11 vs 12+22. Variants showing high pair-wise LD: *CPB2 rs3581419* and *rs3742264* (r^2^ = 0.827), *SEPS1 rs496581* and *rs7178239* (r^2^>0.7), and *SEPS1 rs9874* and *rs7178239* (r^2^>0.7). Detailed information is found in Table S2.

We performed gender-genotype interaction analysis to identify variants that showed different genetic effects in women and men. This test is sensitive to both effect direction and effect size. The variants that gave interaction p-value≤0.01 and were associated with CHD, ischemic stroke or the composite end-point of CVD at p≤0.01 in either women or men in combined analysis of both cohorts are presented in Figure 2 and Table S2. The estimated FDR for the set of all interaction tests with p≤0.01 is 70%, but by using the additional criteria of association p-value≤0.01 in at least one of the genders, the actual FDR is likely to be smaller. The gender-genotype interaction analysis supports our previous findings for *USF1* and *SEPS1* variants in which the disease risk was limited to women [30], [31], providing a gender-genotype interaction p-values<0.01 for the *USF1* variant *rs2774279* and for two *SEPS1* variants, *rs4965814* and *rs9874*. For the *USF1* variant *rs2774279*, the results were also at p<0.05 for women in each cohort separately. Furthermore, for *rs2774279* we also found evidence for association when analyzing prevalent female CHD cases in both cohorts combined (odds ratio of 1.58, 95% CI 1.04–2.40, p = 0.03). We identified variants in additional genes which showed gender-genotype interaction: *CPB2* and coagulation factor XIII, A1 polypeptide (*F13A1*) conferred gender-specific risk in women for CHD, another variant in *CPB2* conferred risk for CVD, and *F5* for ischemic stroke; and for men, interleukin 6 (*IL6*) for CVD. The data obtained with *F5* variant *rs970741* is based on relatively small groups, with only 12 women incident stroke cases carrying the protective allele, and the result should be interpreted with caution. Genotype-cohort interaction analysis showed that none of the gender-specific results emerge from a strong effect in only one of the cohorts but rather both cohorts contribute to the result. For purpose of future meta-analyses, we provide data for all variants analyzed in both cohorts showing genotype-specific hazard ratios for men and women separately and number of individuals and person years in each genotype group (Tables S3a–c).

We tested which of the variants conferring a CHD risk at p <0.05 were also associated with CHD in the prevalent cases. In addition to *USF1* variant *rs2774279*, also the T allele of variant *rs2073658* of *USF1* conferred risk in both incident and prevalent female cases (HR = 1.62, 95% CI 1.04–2.52, p = 0.03 for incident cases, and odds ratio = 1.87, 95% CI 1.26–2.76, p = 0.002 for prevalent cases, additive model, T risk allele). A variant in the *APOBEC2* gene, *rs2395754*, was associated with CHD in both prevalent and incident male cases (HR = 1.45, 95% CI 1.04–2.02, p = 0.03 for incident cases, and odds ratio = 1.43, 95% CI 1.06–1.94, p = 0.02 for prevalent cases, C allele homozygotes compared to T allele carriers).

### Quantitative trait analysis results

We tested whether any of the 172 variants was associated with the CVD-related quantitative traits: total cholesterol, HDL-C, LDL-C, triglycerides, CRP, BMI, WHR and mean blood pressure, analyzing the sub-cohort individuals without a history of CVD at baseline examination. The results showing association in the combined data analyses of both genders at significance level of p<0.01 are shown in Table 5. The estimated FDR for the set of all association tests (including tests for time-to-event responses) with p≤0.01 is 53%. We identified 3 variants displaying effect differences between the cohorts using genotype-cohort interaction analysis (interaction p-value <0.05), and they were removed. *APOE* variant *rs440446*, conferring risk for CHD in time-to-event analysis (Table 4), was associated with triglyceride values, and *FGA* variant *rs2070018* was associated with mean blood pressure, with heterozygotes having the highest blood pressure values. None of the other variants associated with CHD, ischemic stroke, or CVD at p≤0.01 in women and men combined, was associated at p<0.01 with the quantitative traits tested here. However, we identified several interesting associations with each of the traits studied, as discussed below.

**Table 5:** Results with p<0.01for associations between variants and quantitative traits as measured at baseline examination in sub-cohort subjects free of CVD at baseline, women and men combined.

Gene	Variant rs#	Minor/major allele	Model^a^	P^b^	Measured mean value			Number of individuals		
					hom (minor)	HET	hom (major)	hom (minor)	HET	hom (major)
**Total cholesterol** (mmol/l)										
*LCT*	*rs4988235* c	G/A	add	0.002	5.80	5.70	5.48	110	243	159
*LCT*	*rs6719488* c	G/T	add	0.003	5.93	5.71	5.55	57	215	239
*LPIN1*	*rs1050800*	T/C	dom	0.008	5.68	5.48	5.73	11	150	352
*PROC*	*rs5937*	A/G	dom	0.007	5.51	5.58	5.76	41	208	256
**HDL cholesterol** (mmol/l)										
*APOA5*	*rs619054*	A/G	add	0.0006	1.42	1.36	1.29	40	170	304
*F5*	*rs7542281*	T/C	add	0.007	1.25	1.28	1.38	61	226	228
*FUT3*	*rs11673407*	G/A	dom	0.008	1.38	1.35	1.27	64	248	197
*LPIN1*	*rs2577262*	A/G	rec	0.009	1.43	1.30	1.29	74	242	197
**LDL cholesterol** (mmol/l)										
*APOA5*	*rs619054*	A/G	add	0.01	3.23	3.60	3.67	40	163	295
*APOE*	*rs7412*	T/C	dom	0.0003	2.43	3.18	3.64	1	38	445
*LCT*	*rs4988235* c	G/A	add	0.002	3.76	3.63	3.48	106	233	156
*LCT*	*rs6719488* c	G/T	add	0.0008	3.93	3.64	3.52	56	209	229
**Triglycerides** (mmol/l)										
*APOA5*	*rs3135506*	C/G	dom	0.001	2.53	2.01	1.71	2	85	616
*APOA5*	*rs2072560*	T/C	dom	0.0004	3.25	2.01	1.67	4	113	567
*APOE*	*rs440446*	C/G	add	0.005	1.65	1.59	1.89	52	281	359
*F5*	*rs2269648* c	T/C	rec	0.005	1.32	1.82	1.76	44	270	358
*THBD*	*rs6082986*	G/A	rec	0.001	1.44	1.80	1.73	65	313	317
**C-reactive protein** (mg/l)										
*APOE*	*rs429358*	C/T	add	0.002	1.50	2.50	3.75	22	172	413
*CRP*	*rs1800947*	G/C	dom	0.0001	0.28	1.92	3.54	3	74	554
*F5*	*rs9332575*	C/T	rec	0.003	8.00	2.86	3.14	12	118	496
*FGB*	*rs1044291*	T/C	rec	0.008	7.79	2.39	3.43	28	224	362
**Mean blood pressure** (mm Hg)										
*FGA*	*rs2070018*	G/A	add	0.008	113	117	112	13	139	407
*FGG*	*rs1049636*	G/A	dom	0.003	115	115	112	88	267	203
**Body mass index** (kg/m^2^)										
*F13A1*	*rs3024319*	C/G	rec	0.002	28.7	27.3	27.7	110	310	273
*F5*	*rs9332575*	C/T	rec	0.008	30.9	27.4	27.7	12	133	554
*FUT3*	*rs11673407*	G/A	add	0.009	26.6	27.6	28.1	86	331	279
*ICAM1*	*rs3093032*	T/C	rec	0.005	25.1	27.9	27.7	16	170	498
**Waist/hip ratio**										
*CRP*	*rs1130864*	A/G	add	0.002	0.899	0.915	0.916	88	302	311
*F13A1*	*rs3024319*	C/G	rec	0.007	0.926	0.911	0.914	110	310	273
*F5*	*rs9332640*	G/C	rec	0.002	0.895	0.919	0.922	137	365	193
*FUT3*	*rs874232*	C/T	add	0.003	0.911	0.91	0.922	124	355	217
*FUT3*	*rs11673407*	G/A	add	2.00E-05	0.885	0.912	0.926	86	331	279

a For most variants, showing results for additive model (add). Exception is for trait/variant combination in which one of the genotyping groups has <5 individuals, for which the comparison is made between minor allele carriers and non-carriers. Also, showing variants for which the association at p<0.01 is obtained only for minor allele dominant (dom) or recessive (rec) model.

b covariates used in analysis: age at baseline, geographic region, cohort, and gender.

c These variant pairs show similar results due to high LD (r^2^>0.96): *F5 rs2269648* and *rs6029*, *LCT rs4988235* and *rs182549*, *LCT rs6719488* and *rs2236783*.

The strongest association identified for quantitative traits in the combined analysis of women and men was for fucosyltransferase 3 (*FUT3*) variant *rs11673407* and WHR. For men the additive model gave a p-value = 0.00006; for women the association was weaker, but in the same direction (p = 0.07). Haplotype analysis for WHR in men using the *FUT3* variants *rs874232*, *rs778986*, and *rs11673407* identified haplotype CAG as the only one associated with WHR, compared to base haplotype TAA (p = 0.00008) (Table S4a), suggesting that the true causal variant is not one of these 3 variants. Another strong association was found for a rare synonymous *CRP* variant, *rs1800947*, and CRP levels in men (p = 0.0001, recessive model).

The *LCT* variants were associated with total cholesterol and LDL-C in the combined data: The lactase non-persistence genotype (defined as minor allele homozygotes for variant *rs4988235*) was associated with higher cholesterol values. Similarly to *FUT3* variant, the association was stronger for men (for total cholesterol, p = 0.003 and p = 0.005 for variants *rs4988235* and *rs6719488*, respectively, and for LDL-C p = 0.002, and p = 0.0005 for variants *rs4988235* and *rs6719488*, respectively), and in females the association was weaker but in the same direction. Haplotype analysis using the 3 haplotype-tagging variants *rs2304371*, *rs6719488*, and *rs4988235* for men implied that haplotype GGG, tagged by the G allele of variant *rs2304371* was the one associated with both traits, p = 0.003 for total cholesterol and p = 0.005 for LDL-C (compared to base haplotype ATA) (Table S4b). Sub-cohort men homozygotes for the G allele of *rs2304371* have the highest LDL-C values, 4.02 mmol/l (n = 14), compared to 3.74 for GA genotype (n = 109) and 3.55 for AA genotype (n = 243), p = 0.014 for the additive model. Variants *rs6719488* and *rs2304371* are located in the *LCT* gene itself, while the lactase non-persistence variant is located at 14 kb distance upstream of the *LCT* gene. The *LCT* locus on chromosome 2q21.3 is known for being strongly selected during human evolution, with the lactase persistence allele varying in frequency in different populations and even between geographic regions [55]. We observed no differences in allele frequencies of the lactase persistence genotype in the geographic regions studied here (*G* allele frequency 0.46 in Western Finland and 0.44 in Eastern Finland).

Variants that showed different effects on CVD-related quantitative traits in women and men are shown in Table 6, using an interaction p-value cut off ≤0.01 and an association cut off p<0.01 in either women or men in combined analysis of both cohorts. The estimated FDR for the set of all interaction tests with p≤0.01 is 70%, but the additional criteria of association p-value<0.01 in at least one of the genders makes the actual FDR smaller than the upper limit of 70%. As for the disease risk, also here variants in different genes were associated with the traits in women and men. In women, variants in the fibrinogen genes (*FGA* and *FGG*) were associated with HDL-C. Interestingly, none of the genes that are in lipid pathways were associated with lipid variables in women at p<0.01. For weight-related variables, variants that showed gender-specific effect were identified only in women. *USF1* variant *rs2774279*, which was associated with CHD and CVD risk, was also associated with BMI in women, though risk allele carriers had lower BMI. Women with the risk allele also had lower values of CRP. Three variants in *ICAM1* gene associated with WHR in women. Haplotype analysis did not reveal any ICAM1 haplotypes associated more strongly with the trait than single alleles. The largest number of gender-genotype interactions was identified for CRP levels in females.

**Table 6:** Results with p<0.01 for associations between variant and quantitative traits as measured at baseline examination, for variants showing evidence of gender-genotype interaction (p≤0.01), women and men of the sub-cohort free of CVD at baseline.

					Women					Men				
					p-value^c^	Measured mean value			Number of individuals	p-value^c^	Measured mean value			Number of individuals
Gene	Variant rs#	Minor/major allele	Model^a^	p interaction^b/c^		hom (minor)	HET	hom (major)			hom (minor)	Het	hom (major)	
**Total cholesterol** (mmol/l)														
*LPIN1*	*rs893346*	G/A	dom	0.004	0.08	5.15	5.55	5.79	4/24/101	**0.006**	6.78	5.83	5.57	4/83/296
**HDL cholesterol** (mmol/l)														
*FGA*	*rs2070006*	T/C	add	0.0001	**0.0002**	1.73	1.53	1.38	24/75/33	0.2	1.24	1.23	1.29	82/194/105
*FGG*	*rs1800792*	C/T	add	0.01	**0.004**	1.38	1.46	1.62	7/63/62	1	1.2	1.28	1.24	44/150/189
**LDL cholesterol** (mmol/l)														
*APOBEC2*	*rs2395754*	T/C	rec	0.008	0.3	3.73	3.4	3.72	25/63/42	**0.001**	3.35	3.69	3.71	72/192/106
**Triglycerides** (mmol/l)														
*F5*	*rs9332618*	A/G	add	0.0009	0.05	1.64	1.67	1.35	6/59/122	**0.002**	1.28	1.69	1.93	13/142/351
**C-reactive protein** (mg/l)														
*F5*	*rs7542281* d	T/C	dom	0.01	**0.007**	5.88	4.2	2.52	17/71/83	0.8	3.96	2.54	3.26	62/203/193
*LPIN1*	*rs2716610*	T/C	dom	0.003	**0.007**	10.47	7.53	3.44	3/26/142	0.2	1.15	2.03	3.25	3/78/377
*THBD*	*rs1962*	C/T	add	0.004	**0.005**	2.63	2.39	4.99	5/47/119	0.3	3.38	3.45	2.81	14/127/313
*USF1*	*rs2774279*	T/C	add	0.0004	**0.002**	8.87	4.15	3.04	21/67/83	0.06	2.29	2.91	3.05	35/177/240
**Mean blood pressure** (mm Hg)														
*SERPINE1*	*rs2227631* d	G/A	add	0.01	0.3	115	113	111	40/58/33	**0.002**	110	114	116	97/186/129
**Body mass index** (kg/m^2^)														
*ACE*	*rs4320*	A/G	add	0.0009	**0.003**	29	27.8	26.5	36/87/65	0.2	27.5	27.6	28.1	96/246/165
*F13A1*	*rs2274393*	A/G	rec	0.01	**0.005**	31.1	26.5	28	12/66/105	0.8	27.6	28.2	27.4	35/178/291
*FGG*	*rs1800792*	C/T	add	0.002	**0.0005**	30.8	28.2	26.7	12/85/94	0.9	27.9	27.6	27.7	55/209/247
*USF1*	*rs2774279*	T/C	add	0.001	**0.002**	29.5	28.2	26.7	21/77/90	0.2	27	27.7	27.8	38/200/269
**Waist/hip ratio**														
*ICAM1*	*rs281432* d	G/C	add	0.001	**0.006**	0.811	0.807	0.845	43/94/51	0.09	0.956	0.951	0.94	86/262/142
*ICAM1*	*rs5030352* d	G/C	add	0.002	**0.008**	0.814	0.804	0.844	37/92/56	0.1	0.958	0.953	0.942	74/242/172
*ICAM1*	*rs3093030*	T/C	rec	0.005	**0.008**	0.845	0.81	0.817	29/92/70	0.3	0.943	0.951	0.952	89/245/178

a Regression analysis, using as covariates geographic region, cohort, age.

b Interaction p-value tests the null hypothesis that the genotype effect in regression analysis in men and women does not differ from each other.

c Uncorrected p-values.

d High pair-wise LD: r^2^ = 0.80 between *ICAM1 rs281432* and *rs5030352*, r^2^ = 0.97 *F5 rs2227245* and *rs7542281* therefore results for *rs2227245* not shown.

For men, the *APOBEC2* variant *rs2395754*, which associated with CHD in both incident and prevalent cases, was also associated with cholesterol variables. Men carrying the risk allele had higher levels of LDL-C, p = 0.001. In men also a variant in the serpin peptidase inhibitor, clade E member 1 gene was associated with mean blood pressure. In addition to these findings, very few male-specific results at p<0.01 were identified, as shown in Table 6. The strongest associations with lipids for men were for variants that also showed the same trend in women, as discussed above.

## Discussion

The hormonal environment as well as tissue specific gene expression is known to differ significantly between the genders in vertebrates. For many human diseases, gender-dependent differences in the progression and extent of disease have been explained by sex hormones. These hormones may differentially affect gene expression in somatic tissues, thus leading to the gender specific susceptibility to disease [56]. Also for cardiovascular disease, critical determinants of gender differences are sex steroid hormones and their receptors [57]. They interact with and activate, together with other proteins, genes that are possibly involved in CVD pathogenesis in the endothelial and smooth muscle cells [2], [57]. Sex steroid hormones are also expressed in the liver and regulate lipid levels, mostly through hepatic effects on lipoprotein metabolism [57].

Although women and men differ in various aspects related to CHD and ischemic stroke [2]–[4], [6], [7], the difference in genetic effects on disease and its risk factors between women and men remains largely unexplored territory [12]. Recent genome-wide association studies also do not address this issue [14]–[17], [19], [58]. In this candidate gene study we explored the genetic risk profiles for CHD, ischemic stroke and the composite end point of CVD in men and women, as well as the effect of the specific genetic variants on CVD-related quantitative risk factors. Our case-cohort study was based on two prospective cohorts from the relatively homogeneous Finnish population, the sub-cohort representing a random subsample of the original cohort. Detailed information on CVD risk factors recorded before the occurrence of CVD events allowed us to control for confounding factors, such as smoking, lipid levels, blood pressure and obesity, while the inclusion of two separate cohorts allowed for the verification of results. We identified variants in several genes as conferring disease risk for both men and women jointly, while other variants showed evidence for a gender-specific effect. We also identified variants that were associated with quantitative CVD risk factors in both men and women combined, and other variants that showed evidence for gender-genotype interaction. A recent review of gender differences in genetic effects has suggested three criteria for appropriately documented gender differences: (1) The genetic effect is based on the same genetic contrast in both genders; (2) Different genetic subsets in the 2 genders are not compared; and (3) Evidence for a nominally statistically significant gender-gene interaction exists [59]. Our study fulfils all these criteria for the genetic variants showing different effects in men and women. However, studies that replicate these results in larger study samples would be required to confirm or refute the gender-specific associations presented here. With pooling of information across the latest genome-wide association studies [14], [16], [17], [19], [28], there is ample opportunity to test for the presence of gender-genotype interactions behind CHD and ischemic stroke at a genomic level.

In this study, we identified variants in *CPB2*, *F13A1* and *LPIN1* as contributing to female-specific risk for CHD and/or CVD, in addition to a variant in USF1 which we have previously reported [31]. Other variants of *USF1* have also been reported as showing significant gender-genotype interaction for triglycerides and BMI in familial combined hyperlipidemia families [60]. For ischemic stroke, we identified a variant in *F5* as conferring gender-specific risk, in addition to our previously reported association between *SEPS1* variants and ischemic stroke in women [30]. Importantly, we identified a larger number of gender-specific effects for women than for men. For men, only one variant in *IL6* gene was associated with CVD at p≤0.01 and interaction p<0.01. The asymmetry in positive results is similar to a previous large scale candidate gene study of the metabolic syndrome, in which genetic effects were stronger in women [27]. This is also consistent with the larger heritability estimates for stroke and several CVD-related traits in women [11], [13]. These results suggest that genetic effects on CVD risk may be more readily detectable in women, while for men the genetic effects are more confounded by environmental/lifestyle risk factors.

The most consistent result we identified when analyzing women and men jointly was for a variant in the *F12* gene, *rs1801020*. This promoter variant is located in the untranslated exon 1 of the gene, and the T allele was found to be less common in patients with acute coronary syndrome compared to patients with stable coronary artery disease [61]. In our study sample, in which the A ( = T) allele was associated with risk of CHD and CVD, the study setting was very different, and therefore the results are not readily comparable. Variants in the *F12* gene were not present in the Affymetrix 500K and Illumina 300K chips that have been used for the recent genome-wide association studies. The strongest association for quantitative trait variable was between WHR and an intronic variant of the *FUT3* gene, *rs11673407*. The associated variant is not one of the four variants previously associated with Lewis blood phenotype [62] (*rs778986* studied here) and which have been reported to be associated with several CVD-related risk factors [63].

Two of the genes we selected to this study, *LCT* and *APOBEC2*, have not been previously associated with molecular pathogenesis of cardiovascular disease. We found association between *LCT* variants and both total and LDL cholesterol. Haplotype analysis implied that the associated variants are in the *LCT* gene itself, and not necessarily related to the lactase persistence variant upstream of the gene. The *C* allele of the exonic variant *rs2304371*, which was associated with highest cholesterol values, is the ancestral allele, present in other mammals and located in a highly conserved region. We also found that a variant in *APOBEC2* conferred risk of CHD in men and was associated with higher levels of LDL-C. *APOBEC2* belongs to the cytidine deaminase superfamily, and is closely related to *APOBEC1* [64]. APOBEC1 mediates the editing of apolipoprotein B mRNA [65]. *APOBEC2* is expressed exclusively in heart and skeletal muscle [64], and its function is still largely unknown.

To summarize, we have identified several variants of relevant candidate genes that may confer risk of CHD, ischemic stroke or CVD and/or associate with quantitative CVD-risk factors in a gender-specific manner, and other variants which probably confer risk in both women and men. The identified disease associations and quantitative trait associations had uncorrected p-values≤0.01 for both genders combined and on the basis of the FDR analysis we expect that half of the findings are true positives. For interaction analysis, we may expect that at least third of the reported results are true positives. However, the FDR analysis for the interaction analysis is conservative, because it does not account for the additional criteria we used of association p-value<0.01 for the trait itself in either men or women. Thus, we are convinced that some of the results represent a real effect of variants on disease/trait, but obviously require replication in other studies. In addition, our study had low power to detect genetic effects with HR<1.8 or coefficient<0.3, thus some of the variants we have studied that show no genetic effect might represent false negative results. The possible differences in genetic risk profiles between the genders should be addressed in more detail in genetic studies of CVD, and more focus on female CVD risk is warranted also in genome-wide association studies.

## Supporting Information

Table S1 The genetic variants analyzed in the current study
(0.09 MB XLS)

Table S2 Gender-specific results (p≤0.01, uncorrected), in which there was gender-genotype interaction (p≤0.01, uncorrected) in coronary heart disease, ischemic stroke and cardiovascular disease
(0.06 MB DOC)

Table S3 Genotype association results for women and men (uncorrected p-values) for coronary heart disease (a), ischemic stroke (b) and cardiovascular disease (c)
(0.27 MB XLS)

Table S4 (a) Haplotype analysis for body mass index (BMI) and waist/hip ratio (WHR) for FUT3 variants, sub-cohort men free of CVD at baseline; (b)Haplotype analysis for total cholesterol and LDL cholesterol for LCT variants, sub-cohort men free of CVD at baseline.
(0.05 MB DOC)

Figure S1 Power simulations for time-to-event analysis for risk allele frequencies of 0.2 and 0.4, combining both cohorts, using p-value cut-off of 0.01 and assuming for interaction analysis (IA) no effect for men while testing different effect values for women. The lines connect different value points and are not interpolations. HR = hazard ratio.
(7.13 MB TIF)

Figure S2 Power simulations for quantitative trait analysis using BMI as an example, testing risk allele frequencies of 0.2 and 0.4, combining both cohorts, using p-value cut-off of 0.01 and assuming for interaction analysis (IA) no effect for men while testing different effect values for women. The lines connect different value points and are not interpolations. Regression coefficients are given in standard deviation scale. BMI = body mass index.
(7.13 MB TIF)
