# Supplementary material for: Gender Differences in Genetic Risk Profiles for Cardiovascular Disease
Source: PLoS One. 2008 Oct 31;3(10):e3615. doi: 10.1371/journal.pone.0003615 (PMC2574036; doi:10.1371/journal.pone.0003615)
Supplement: Table S2 — Gender-specific results (p≤0.01, uncorrected), in which there was gender-genotype interaction (p≤0.01, uncorrected) in coronary heart disease, ischemic stroke and cardiovascular disease (0.06 MB DOC) [file pone.0003615.s002.doc]

**Table S2.** Gender-specific results (p≤0.01, uncorrected), in which there was gender-genotype interaction (p≤0.01, uncorrected) in coronary heart disease, ischemic stroke and cardiovascular disease

|  |  |  |  | **allele1/** | **Minor** | **Interaction** | **Women** | |  | **Men** | | **Allele 1 freq case/control** | | | |
| --- | --- | --- | --- | --- | --- | --- | --- | --- | --- | --- | --- | --- | --- | --- | --- |
| **phenotype** | **model^a^** | **Gene** | **rs#** | **allele2** | **allele** | **p-value^b/c^** | **p-value** | **HR (95% CI)** |  | **p-value** | **HR (95% CI)** | **women** | | **men** | |
| Coronary heart disease | dominant | *CPB2* | *rs3742264* ^c^ | C/T | T | 0.009 | **0.0006** | 0.31 (0.16 - 0.60) | 0.6 | | 0.89 (0.58 - 1.38) | 0.51/0.61 | | 0.58/0.60 | |
|  | dominant | *F13A1* | *rs2295752* | A/C | A | 0.004 | **0.004** | 2.21 (1.29 - 3.79) | 0.4 | | 0.88 (0.63 - 1.22) | 0.33/0.26 | | 0.27/0.31 | |
|  | additive | *USF1* | *rs2774279* | C/T | T | 0.007 | **0.004** | 1.91 (1.23 - 2.98) | 0.6 | | 0.94 (0.73 - 1.21) | 0.78/0.68 | | 0.73/0.73 | |
| Ischemic stroke | recessive | *F5* | *rs970741* | A/G | G | 0.01 | **0.01** | 2.64 (1.23 - 5.69) | 0.5 | | 0.84 (0.52 - 1.35) | 0.86/0.78 | | 0.78/0.80 | |
|  | dominant | *SEPS1* | *rs4965814* ^c^ | C/T | C | 0.003 | **0.002** | 2.89 (1.48 - 5.65) | 0.5 | | 0.86 (0.54 - 1.38) | 0.37/0.20 | | 0.24/0.24 | |
|  | dominant | *SEPS1* | *rs9874* ^c^ | C/T | C | 0.007 | **0.0009** | 3.32 (1.64 - 6.73) | 0.9 | | 1.02 (0.63 - 1.66) | 0.28/0.13 | | 0.16/0.17 | |
| Cardiovascular disease | recessive | *CPB2* | *rs17067700* | A/G | A | 0.01 | **0.01** | 2.69 (1.22 - 5.90) | 0.3 | | 0.71 (0.38 - 1.33) | 0.28/0.28 | | 0.31/0.29 | |
|  | recessive | *IL6* | *rs2069840* | C/G | G | 0.004 | 0.1 | 1.50 (0.93 - 2.43) | **0.006** | | 0.66 (0.49 - 0.88) | 0.76/0.75 | | 0.75/0.79 | |
|  | additive | *LPIN1* | *rs10192566* | C/G | G | 0.01 | **0.006** | 1.62 (1.15 - 2.28) | 0.7 | | 0.97 (0.78 - 1.20) | 0.59/0.50 | | 0.53/0.55 | |
|  | additive | *USF1* | *rs2774279* | C/T | T | 0.007 | **0.004** | 1.75 (1.20 - 2.57) | 0.6 | | 0.94 (0.75 - 1.18) | 0.76/0.68 | | 0.73/0.73 | |
| **^a^**Time-to-event analysis, using as covariates geographic region, cohort, HDL-cholesterol, non-HDL cholesterol, body mass index, hypertension, smoking status, history of diabetes. Multiplicative model: 11>12>22, dominant model: 11+12 vs 22, recessive model: 11 vs 12+22 | | | | | | | | | | | | | | | |
| **^b^**Interaction p-value tests the null hypothesis that the genotype effect in time-to-event analysis in men and women does not differ from each other. | | | | | | | | | | | | |  | |  |
| ^c^ Pair-wise LD: r^2^=0.827 between *CPB2 rs35814191* and *rs3742264*, r^2^>0.7 between *SEPS1 rs496581* and *rs7178239*, and *rs9874* and *rs7178239* | | | | | | | | | | | | |  | |  |
