# Supplementary material for: Gender Differences in Genetic Risk Profiles for Cardiovascular Disease
Source: PLoS One. 2008 Oct 31;3(10):e3615. doi: 10.1371/journal.pone.0003615 (PMC2574036; doi:10.1371/journal.pone.0003615)
Supplement: Table S4 — (a) Haplotype analysis for body mass index (BMI) and waist/hip ratio (WHR) for FUT3 variants, sub-cohort men free of CVD at baseline; (b)Haplotype analysis for total cholesterol and LDL cholesterol for LCT variants, sub-cohort men free of CVD at baseline. (0.05 MB DOC) [file pone.0003615.s004.doc]

**Table S4a.** Haplotype analysis for body mass index (BMI) and waist/hip ratio (WHR) for *FUT3* variants, sub-cohort men free of CVD at baseline

|  | ***rs874232*** | ***rs778986*** | ***rs11673407*** |  | **Haplotype p-value** | |
| --- | --- | --- | --- | --- | --- | --- |
| **Haplotype** |  |  |  | **Freq** | **BMI** | **WHR** |
| 1^a^ | T | A | A | 0.26 | NA | NA |
| 2 | C | A | G | 0.27 | 0.01 | 0.00008 |
| 3 | T | G | A | 0.22 | 0.67 | 0.90 |
| 4 | C | A | A | 0.18 | 0.13 | 0.81 |
| 5 | T | A | G | 0.06 | 0.21 | 0.40 |
| Global |  |  |  |  | 0.14 | 0.0003 |
| ^a^Haplotype 1 used as base-haplotype to which all comparisons are made | | | | | | |

**Table S4b.** Haplotype analysis for total cholesterol and LDL cholesterol for *LCT* variants, sub-cohort men free of CVD at baseline

|  | | | | | | |
| --- | --- | --- | --- | --- | --- | --- |
|  | ***rs2304371*** | ***rs6719488*** | ***rs4988235*** |  | **Haplotype p-value** | |
| **Haplotype** |  |  |  | **Freq** | **Total chol** | **LDL-chol** |
| 1^a^ | A | T | A | 0.50 | NA | NA |
| 2 | G | G | G | 0.19 | 0.003 | 0.005 |
| 3 | A | T | G | 0.18 | 0.100 | 0.135 |
| 4 | A | G | G | 0.12 | 0.151 | 0.147 |
| Global |  |  |  |  | 0.018 | 0.030 |
| ^a^Haplotype 1 used as base-haplotype to which all comparisons are made | | | | | | |
