# Supplementary figures and images for: Gender Differences in Genetic Risk Profiles for Cardiovascular Disease
Source: PLoS One. 2008 Oct 31;3(10):e3615. doi: 10.1371/journal.pone.0003615 (PMC2574036; doi:10.1371/journal.pone.0003615)

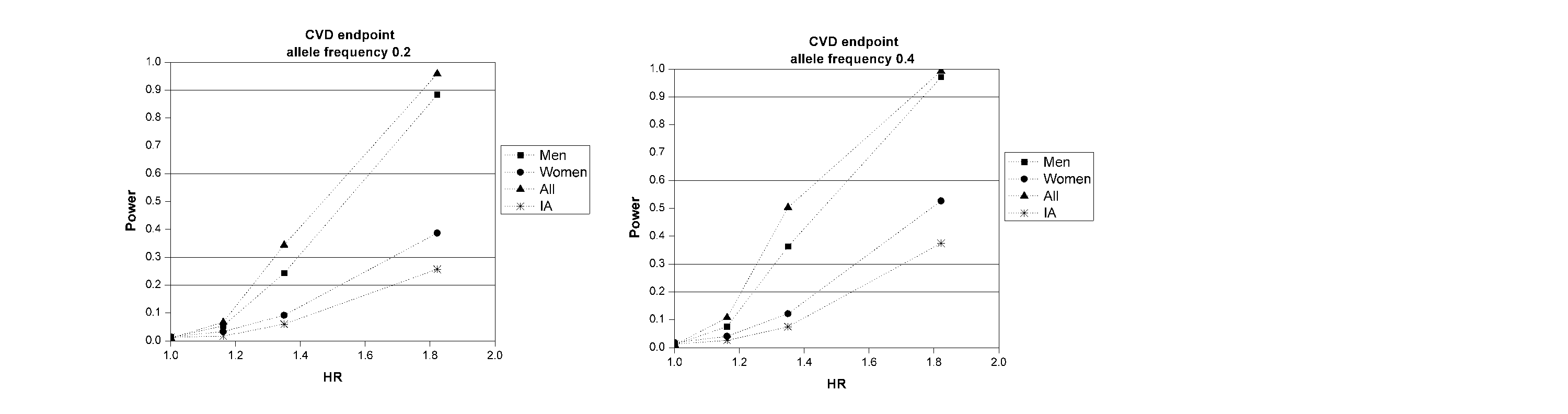

Supplement: Figure S1 — Power simulations for time-to-event analysis for risk allele frequencies of 0.2 and 0.4, combining both cohorts, using p-value cut-off of 0.01 and assuming for interaction analysis (IA) no effect for men while testing different effect values for women. The lines connect different value points and are not interpolations. HR = hazard ratio. (7.13 MB TIF) [file pone.0003615.s005.tif]

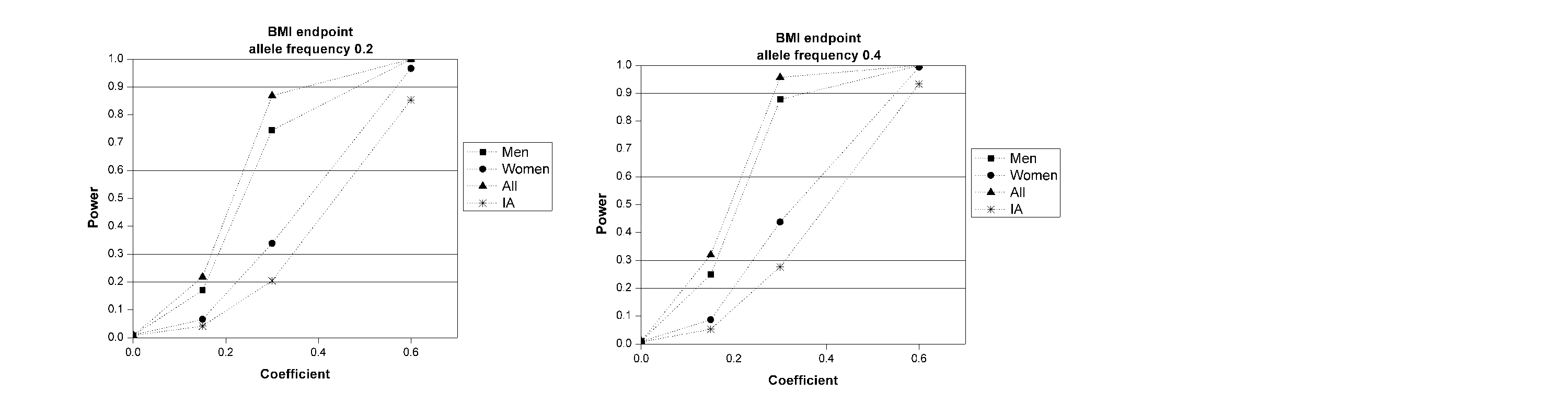

Supplement: Figure S2 — Power simulations for quantitative trait analysis using BMI as an example, testing risk allele frequencies of 0.2 and 0.4, combining both cohorts, using p-value cut-off of 0.01 and assuming for interaction analysis (IA) no effect for men while testing different effect values for women. The lines connect different value points and are not interpolations. Regression coefficients are given in standard deviation scale. BMI = body mass index. (7.13 MB TIF) [file pone.0003615.s006.tif]
